# Effects of overground gait training assisted by a wearable exoskeleton in patients with Parkinson’s disease

**DOI:** 10.1186/s12984-023-01280-y

**Published:** 2023-11-16

**Authors:** Virginie Otlet, Clémence Vandamme, Thibault Warlop, Frédéric Crevecoeur, Renaud Ronsse

**Affiliations:** 1 Institute of Mechanics, Materials, and Civil Engineering, UCLouvain, Louvain-la-Neuve, Belgium; 2 Institute of Neuroscience, UCLouvain, Brussels, Belgium; 3 Louvain Bionics, UCLouvain, Louvain-la-Neuve, Belgium; 4 Institute of Information and Communication Technologies, Electronics and Applied Mathematics, UCLouvain, Louvain-la-Neuve, Belgium; 5 Service de Neurologie, Centre Hospitalier de Wallonie Picarde, Tournai, Belgium; 6 Service de Neurologie (Pathologie du Mouvement), Centre Hospitalier Universitaire de Lille, Lille, France

**Keywords:** Long-range autocorrelations, Parkinson’s disease, Walking assistance, Wearable device

## Abstract

**Background:**

In the recent past, wearable devices have been used for gait rehabilitation in patients with Parkinson’s disease. The objective of this paper is to analyze the outcome of a wearable hip orthosis whose assistance adapts in real time to the patient’s gait kinematics via adaptive oscillators. In particular, this study focuses on a metric characterizing natural gait variability, i.e., the level of long-range autocorrelations (LRA) in series of stride durations.

**Methods:**

Eight patients with Parkinson’s disease (Hoehn and Yahr stages 1$-$2.5) performed overground gait training three times per week for four consecutive weeks, assisted by a wearable hip orthosis. Gait was assessed based on performance metrics such as the hip range of motion, speed, stride length and duration, and the level of LRA in inter-stride time series assessed using the Adaptive Fractal Analysis. These metrics were measured before, directly after, and 1 month after training.

**Results:**

After training, patients increased their hip range of motion, their gait speed and stride length, and decreased their stride duration. These improvements were maintained 1 month after training. Regarding long-range autocorrelations, the population’s behavior was standardized towards a metric closer to the one of healthy individuals after training, but with no retention after 1 month.

**Conclusion:**

This study showed that an overground gait training with adaptive robotic assistance has the potential to improve key gait metrics that are typically affected by Parkinson’s disease and that lead to higher prevalence of fall.

*Trial registration*: ClinicalTrials.gov Identifer NCT04314973. Registered on 11 April 2020.

## Introduction

Gait disorders cause major issues for patients with Parkinson’s disease, starting in the early stages of the disease [1]. In particular, patients may have a hypokinetic gait, characterized by a slower gait speed and shorter stride length [2]. These gait disorders are associated with upcoming falls [3]. Indeed, the risk of falling is twice as likely in patients with Parkinson’s disease as in age-matched healthy individuals [4]. This can lead to a fear of falling in some patients, which induces them to decrease their physical activities, and thus affects their independence and quality of life [5].

There exist several physical therapies in order to delay and/or mitigate the impact of these motor disorders, ranging from regular physiotherapy to dance [6]. Taking advantage of advances in research on robot-assisted gait training for other pathologies, the last decade has also seen the emergence of studies on the rehabilitative effects of these therapies on the gait of patients with Parkinson’s disease. In these studies, patients were trained with a robot moving their legs following a stereotyped kinematic pattern. These studies used treadmill exoskeletons, such as the Lokomat^®^ (Hocoma, Zurich, Switzerland), or end-effector systems, such as the Gait Trainer GT1 (Reha-Stim, Berlin, Germany) or the G-EO (Reha Technology, Olten, Switzerland). They showed an increase in gait speed [7–19], in stride length [7, 8, 10, 12, 13, 15–17] and in cadence [8, 12, 13, 17], as well as a decrease in motor symptoms [8, 11–14, 19] and an increase in endurance [9, 16, 18, 19]. Some of these improvements were maintained between 1 and 6 months after training [8, 9, 14, 16]. Some hypotheses on how these therapies influence these gait metrics have been put forward. Firstly, it could act as an external rhythmic cue on which patients can focus, thus compensating for the defective internal rhythm of the basal ganglia. Secondly, the repetition of gait-like movements might enhance the activation of automatic spinal control of locomotion. Finally, robot-assisted gait training also induces an increased physical activity, therefore strengthening the lower-limb muscles of patients as well as their cardiovascular status [20, 21].

More recently, studies have been conducted with wearable exoskeletons that can be used in more ecological environments, such as the hip orthosis SMA (Honda R&D, Tokyo, Japan), or the knee orthosis Keeogo Rehab^™^ (B-Temia, Quebec, Canada). A training of 10 overground sessions with the hip orthosis improved gait endurance, metabolic cost and motor symptoms of patients [22]. On the other hand, with the knee orthosis, patients improved their cognitive and physical functions while wearing it, but they did not increase their gait speed after training [23]. These wearable devices offer the advantage of enabling to study their effects outside a treadmill, which has been shown to significantly influence the way people walk [24]. Moreover, they allow to be used not only in rehabilitation protocols, but also for assistance, since they open the perspective to be worn in everyday life, at least for the most affected patients.

This wearability is particularly interesting in the assessment of the level of long-range autocorrelations (LRA) in series of stride durations. The presence of LRA in these series captures that the duration of the current stride statistically depends on all those that happened in the past [25]. The precise origin of the presence of LRA in the locomotor system is still debated. Several studies hypothesized that it may arise from the complex coordination and interaction of various components and subsystems within this system, acting at different time scales [26, 27]. Moreover, this system being redundant, i.e., its components can be used interchangeably for the same task [27], it is adaptable and robust to both internal and external disturbances, such as minor variations in the walking surface or natural neuromuscular noise [28]. As a complementary perspective to this statement, Dingwell and colleagues proposed the Goal Equivalent Manifold framework [29], which suggests that there are countless ways to modulate a step by varying features such as gait speed, step length, or duration. Humans can therefore adjust their walking features from stride to stride to achieve specific goals while enhancing task performance, such as maintaining constant walking speed on a treadmill [29, 30] or a constant gait cycle timing when walking to the rhythm of a metronome [29].

LRA is thus a key property of biological series and has been proposed as a marker of gait instability in the particular case of locomotion. Indeed, several studies have reported a decreased level of LRA in series of stride durations of elderly walkers [31] and patients with Parkinson’s disease [32] as compared to a control group, reflecting a more random temporal organization of their walking pattern [32, 33]. Moreover, it has been demonstrated that this metrics is influenced by the walking support (i.e., overground vs. treadmill) in patients with Parkinson’s disease, with the treadmill acting like an external pacemaker regulating the leg movement timing [34, 35]. This further highlights the importance of using wearable devices when assessing the presence of LRA in series of stride durations.

Two recent modeling studies [36, 37] predicted that an oscillators-based wearable hip orthosis would increase the level of LRA towards the level of healthy walkers in series of stride durations of patients with Parkinson’s disease. A subsequent study [38] analyzing the effect of such an orthosis on healthy people aged over 55, corresponding to the mean age of onset of Parkinson’s disease [39], showed that it can improve gait metrics such as the hip range of motion, gait speed, stride length and cadence, without impacting the level of LRA. These metrics are precisely among those deteriorated by Parkinson’s disease and are associated with an increased risk of falling [3].

Therefore, the purpose of the present paper is to assess the effects of robot-assisted gait training in patients with Parkinson’s disease, using a wearable device relying on an algorithm adapting in real time to the patient’s kinematics. This study is the first to investigate the effect of an assistance based on adaptive oscillators on patients affected by this disease after overground gait training. This allows measuring the impact of this assistance in a semi-ecological condition, and to leverage this condition to assess a critical marker of gait affected by this disease, i.e., the level of LRA in series of stride durations.

## Methods

### Participants

Eight patients with Parkinson’s disease participated in this study. They were recruited according to the following inclusion criteria: positive diagnosis according to the UK Brain Bank Criteria, modified Hoehn & Yahr (H&Y) scale between 1 and 3, a minimum of 24/30 on the Mini-Mental State Examination (MMSE), and no contraindication to physical exercising. Medication was stable for the 4 weeks preceding the study, and was maintained throughout the study. One participant was treated with Deep Brain Stimulation. The study took place at the Mounier Sports Center (Brussels, Belgium) between February 2022, the date of first inclusion, and November 2022, the date of last follow-up visit. Clinical characteristics and anthropometrics data of patients are displayed in Table 1.

**Table 1:** Characteristics of the study population

Patient	Age	Gender	Weight (kg)	H&Y	Most affected side
#1	76	M	83	2	Left
#2^a^	67	M	79.5	2.5	Right
#3	69	M	70.5	2.5	Left
#4	73	F	53.5	1	Left
#5	57	M	93	2	Right
#6	76	M	83.5	2	Right
#7	72	M	83	2	Left
#8	75	M	79.5	2	Left

H&Y stands for the Hoehn and Yahr scale

^a^Patient implanted with Deep Brain Stimulation

### Procedure

For each patient, the entire protocol lasted 8 weeks. It began with a first evaluation session (T0), consisting in evaluating their motor disorders through the MDS-Unified Parkinson’s Disease Rating Scale (MDS-UPDRS) part III score, also allowing the identification of the side most affected by the disease for each patient, and their cognitive state through the MMSE, both assessed by a neurologist. Then, the balance functions were evaluated using the Balance Evaluation Systems Test (Mini-BESTest), assessed by a physiotherapist. Moreover, patients were asked to walk at their comfortable speed in a sports hall, following a rectangular path of 7 m $\times$ 12 m with rounded corners in order to have the most steady gait for LRA assessment. Walking sessions were performed in a quiet environment so as not to increase the attentional cost of walking [32]. Patients performed several laps during 8 min. Speed steadiness was verified by timing the time taken by the subject to complete each lap, and delivering qualitative instructions to adapt walking speed if needed. During this walking session, patients wore a motion capture system (MVN Awinda, Xsens, Enschede, the Netherlands) composed of 8 inertial measurement units (IMUs), allowing to reconstruct the movement of their hips as explained in "Gait metrics". They also wore 2 IMUs (NGIMU, x-io Technologies, Bristol, UK), placed just above the lateral malleolus of both ankles, with their x-axis oriented in the direction of walking. These were used to obtain the sagittal angular velocities for calculating series of stride durations, as explained in "Stride intervals computation". Finally, patients were asked to complete a questionnaire at home about their confidence in performing daily activities without losing balance, assessed through the Activities-specific Balance Confidence (ABC) scale.

**Fig. 1:**
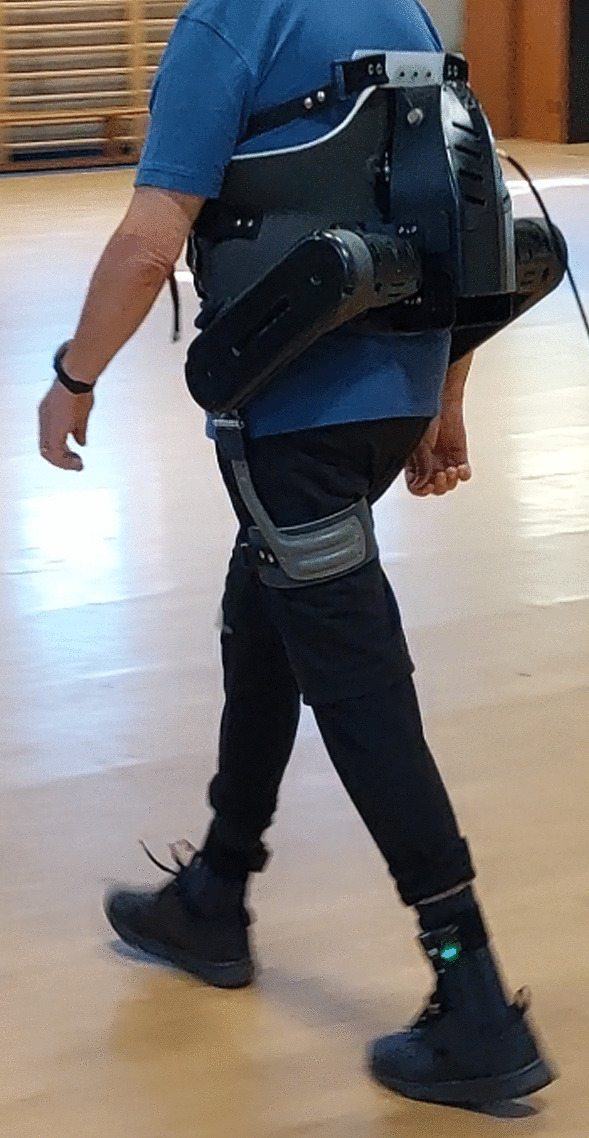
The Active Pelvis Orthosis (IUVO, Pisa, Italy) worn by one of our patients

Thereafter began an intervention phase, consisting of three training sessions a week during 4 weeks, similar to what has already been done in previous studies as summarized in [21]. During these 12 sessions, patients walked with a bilateral wearable Active Pelvis Orthosis (APO, IUVO, Pisa, Italy, Fig. 1) during 5 to 8 min, after a short period where they can adapt their gait to the device’s assistance. This orthosis is controlled by an algorithm relying on adaptive oscillators, such that it continuously synchronizes with the recorded hip trajectories, and adapts to changes in these signals [40]. In brief, this control framework does not impose the patient to follow a prescribed kinematic pattern, but rather delivers a torque that tends to attract the patient’s hips towards their own predicted trajectory, estimated in the future by a prescribed phase lead $\Delta \varphi$. The torque provided by the orthosis is thus given by [41]:

1 $$\begin{aligned} T = k(\hat{x}(\varphi +\Delta \varphi )-\hat{x}(\varphi )), \end{aligned}$$

where *k* is a tunable virtual stiffness [Nm/rad], $\varphi$ is the gait phase estimated by the oscillators [% of gait cycle], $\Delta \varphi$ is the tunable phase lead [% of gait cycle], and $\hat{x}(\varphi )$ [rad] is the hip position estimated by the oscillators (see [42, 43] for further details). In this study, the virtual stiffness was adjusted according to the weight of the subject, i.e., so that the peak torque delivered at the hip was equal to 0.1 Nm/kg, corresponding to a comfortable and safe level of assistance as reported in [44]. This value was determined during the first training session, and then maintained constant throughout the following sessions. The phase lead $\Delta \varphi$ determining how far in advance the signal of the hip is predicted for computing the injected torque was set to 10% of gait cycle.

This intervention phase was followed by a second evaluation session (T1), taking place 1 or 2 day(s) after the last training session. During this session, the same clinical tests as during the first evaluation session were performed, with the exception of the MMSE. This evaluation session was repeated after a 4-week wash-out period (T2).

### Stride intervals computation

The series of stride durations were obtained in the same manner as described in [38]. Briefly, the sagittal shank angular velocity was recorded at a sample rate of 500 Hz using both IMUs, which include a 200 Hz antialiasing low-pass filter on the gyroscope signals. A zero-crossings detection algorithm was used in order to obtain inter-stride time series, i.e., the time between two consecutive heel strikes of the same foot. The maxima of the signal were first identified. Then, the first sign change occurring after each of these maxima was detected. Finally, a linear interpolation was performed between both adjacent points to obtain the most accurate zero crossing detection. When all these events were detected, the inter-stride time series was obtained by differentiating the series of these time-stamped events.

Patients walked between 5 and 8 min for each session, depending on their daily physical condition, fatigue, and their gait speed. The first and last 10 strides of the series were discarded, in order to restrict our analysis to steady-state behavior only, with the objective to keep as many strides as possible, with a minimum of 256 as recommended in [45] for LRA assessment. Only data from the most affected side were analyzed. However, due to connection issues between the IMUs and the computer, some trials displayed gaps in the recorded data. This happened in three of the 24 evaluation sessions. In that case, data from the least affected side were used.

### Gait metrics

Regarding the evaluation sessions, several gait metrics have been computed to study the effect of training on the patient behavior. On the first hand, some spatiotemporal gait metrics were computed. The walking speed per lap was computed by dividing the lap distance (38 m) by the recorded time taken by subjects to walk through each of them. The mean stride duration over each lap was obtained from the inter-stride time series, divided into laps thanks to the average measured time to make a lap. Finally, the average stride length per lap was obtained by taking the product between the stride duration and the walking speed per lap. The stride length and the walking speed were then normalized by the leg length of each subject.

On top of this, the hip motion was reconstructed from the motion capture system signals. The accelerometer and magnetometer signals from each IMUs of the system, recorded at a sample rate of 100 Hz, were used to determine the orientation and position of each IMU relative to that of the pelvis. From these, the movement of each lower-limb segment was obtained and used to derive the hip angle signals, which were low-pass filtered at a cutoff frequency of 18 Hz. Finally, the flexion-extension hip range of motion (ROM) was computed as the difference between the highest and the lowest value of this signal over a gait cycle. As for the series of stride durations, only data from the most affected side were analyzed. Data from two acquisitions could not be reconstructed correctly (subjects #3 in T1 and #6 in T2) and were thus withdrew from the analyses.

### Long-range autocorrelations assessment

Regarding the evaluation sessions, a more complex metric was also extracted from the series of stride durations, i.e., the level of LRA in these series, characterized by the fractal scaling exponent $\alpha$. To compute this exponent, we used the Adaptive Fractal Analysis (AFA). This method is described in details elsewhere [46, 47]. Briefly, the integrated time series of length *N* was divided into overlapping subseries of length *w*. Second order quadratic polynomials were then fitted to each subseries and pasted together to obtain a globally smooth trend signal. The residual variance *F*(*w*) of the difference between this global trend and the original series was reported for several subseries sizes *w*, ranging from 5 to the first power of 2 smaller than *N*/2. To obtain evenly spaced values of *w* in a logarithmic scale, the range of $\log _2(w)$ was divided into a series of intervals of equal length with a step size of 0.5, and the points falling within each interval were averaged. This range of window sizes was determined as the most appropriate to handle non-stationary time series, i.e., with low frequency trends. Finally, the fractal exponent $\alpha$ was obtained as the slope of the linear regression of $\log _2(F(w))$ as a function of $\log _2(w)$. A value of $\alpha > 0.5$ indicates the presence of long-range autocorrelations in inter-stride time series [46].

### Level of assistance

Since the assistive method based on adaptive oscillators constantly adapts to the patient behavior, it is not possible to predict how much mechanical energy will be delivered to the patient during each training session. Therefore, this becomes a metric of interest to be investigated. The orthosis behavior during training sessions was quantified through signals acquired by onboard sensors at 100 Hz. The hip flexion-extension angle was recorded by an absolute encoder, and time-differentiated to obtain the angular velocity. The injected torque was indirectly quantified by measuring the deformation of a torsional spring embedded in the device actuation chain [41]. The torque injected was first normalized by the weight of each subject, then divided into gait cycles using the maximum hip extension angle as separation between cycles. It was then used to compute the energy injected to the hip per cycle [J/kg]:

2 $$\begin{aligned} E = {\int}_{cycle}T \, \dot{x} \textrm{d} t, \end{aligned}$$

with *T* the injected torque [Nm/kg], and $\dot{x}$ the hip angular velocity [rad/s]. The maximal torque injected at the hip per gait cycle was also analyzed.

### Statistical analysis

Data were processed with Matlab version R2019a, and statistical tests were performed in R version 4.2.2. Statistics were performed on the spatiotemporal gait metrics (one data point per lap), on the hip ROM (one data point per gait cycle), and on the clinical scores (one data point per evaluation session). The three evaluation sessions were compared to each other via linear mixed-effects models fitted to the different studied metrics. These include fixed effects, capturing average trends of the metric for each evaluation session, and random effects, capturing the extent to which these trends vary across participants [48]. It is particularly interesting with patients with Parkinson’s disease, who generaly display heterogeneous behavior [49]. The linear mixed-effects model equation is given by:

3 $$\begin{aligned} Y_{i,j} = \gamma _0 + I_{i} + bX_{i,j} + \epsilon _{i,j} , \end{aligned}$$

with $Y_{i,j}$ the gait metric for the *i*th subject and the *j*th repetition (lap or cycle), $\gamma _0$ a general intercept, $I_{i}$ a random intercept for each subject, *b* the regression coefficient for the evaluation sessions, $X_{i,j}$ the evaluation sessions, and $\epsilon _{i,j}$ the residuals. An analysis of variance was then performed on these models, using a Kenward-Roger’s approximation to degrees of freedom [50]. If the *p*-value of this test was lower than 0.05, Tukey’s tests for multiple pairwise comparisons were performed, using the Benjamini-Hochberg correction [51]. The variances of these three sessions were also compared with a Levene’s test [52]. If significant, this test was followed by pairwise Levene’s tests, and a Benjamini-Hochberg correction was applied on the resulting *p*-values.

Linear mixed-effects models were also used to assess whether the evolution of maximal injected torque and injected energy through trainings was significant or not, using the same equation as (3) with $X_{i,j}$ being the training sessions.

For graphical representation, the relative change in spatiotemporal gait metrics and ROM was computed by taking the difference between the values in T1 or T2 and T0, divided by the value in T0 and converted in percentage. For these metrics, inter-subject variability is represented through the standard error of the mean, computed as the standard deviation divided by the square root of the number of subjects.

## Results

**Fig. 2:**
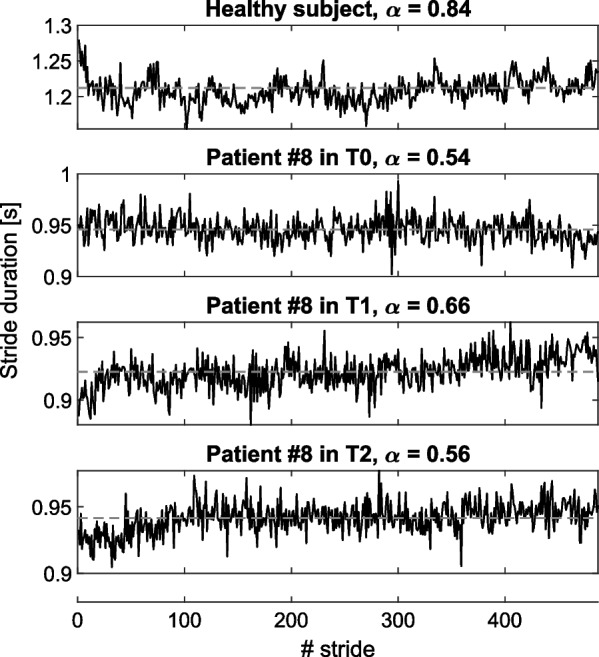
From top to bottom: series of stride durations of a healthy 62-year-old subject freely walking overground during a pilot test, and patient #8 in T0, T1 and T2. The gray dashed lines indicate the mean stride durations, and $\alpha$ is the fractal exponent

**Fig. 3:**
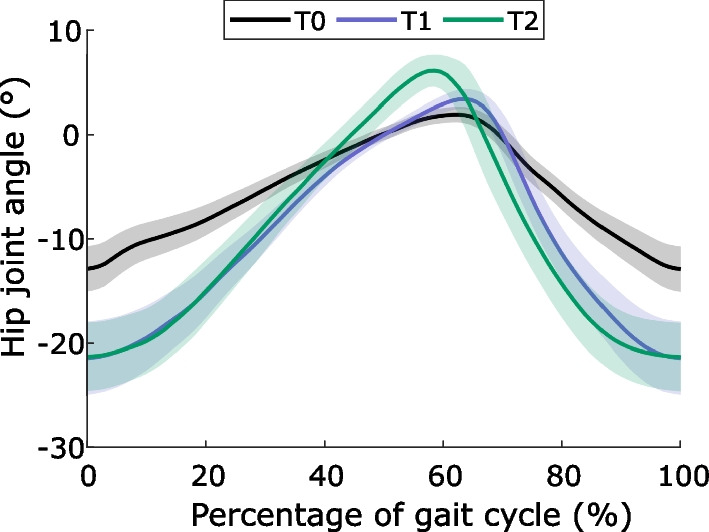
Left hip angle profiles for patient #1 in the three evaluation sessions, with flexion (resp. extension) angles indicated by positive (resp. negative) values. Signals were time-normalized over the gait cycle. Solid lines represent the mean and shaded areas represent the standard deviation over all gait cycles

**Fig. 4:**
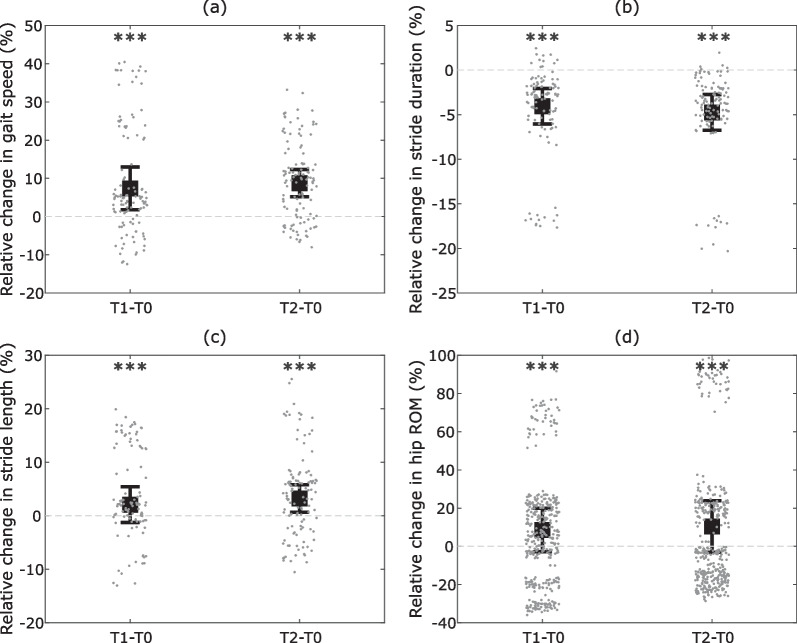
Relative changes in (**a**) normalized gait speed, (**b**) stride duration, (**c**) normalized stride length per lap, and (**d**) hip ROM per gait cycle, for T1 and T2 relative to T0. Squares represent the mean across patients and whiskers indicate standard error of the mean. Each point corresponds to individual data of a given participant in a given lap or gait cycle. Significance level: ***$p \le 0.001$

**Fig. 5:**
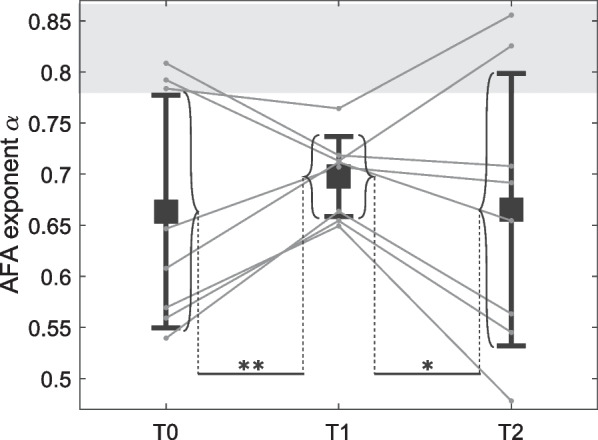
LRA level, characterized by $\alpha$ exponent, for the three evaluation sessions. Each gray line corresponds to the data from one patient. Squares represent the mean across patients and whiskers indicate standard deviation across patients. The shaded area corresponds to values of healthy walkers obtained by applying AFA on 10 series of 1024 points from [53]. Brackets indicate significant differences between standard deviation. Significance levels: **$p \le 0.01$, *$p \le 0.05$

**Fig. 6:**
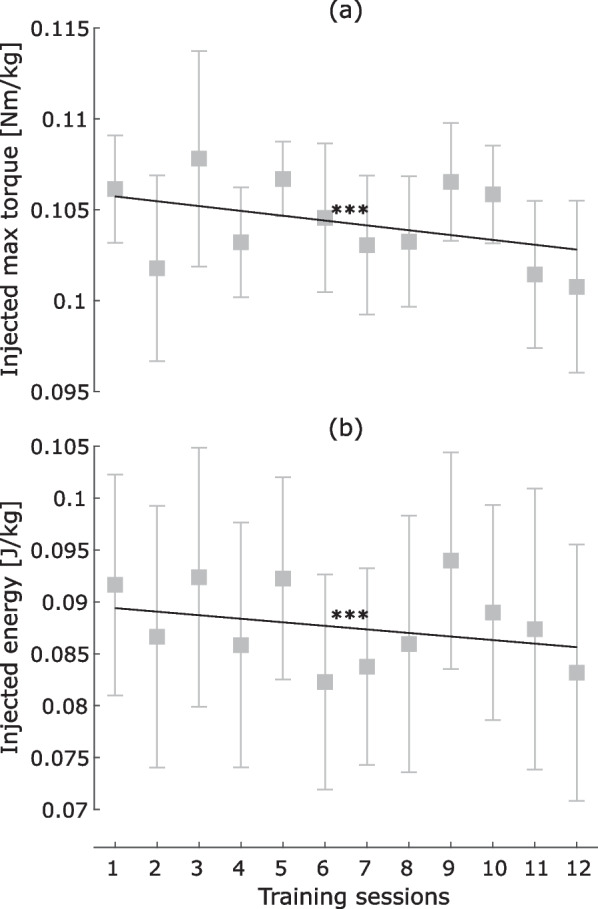
Evolution of the injected (**a**) maximal torque and (**b**) energy at the hip during training sessions. Squares represent the mean across participants and whiskers indicate standard error of the mean. Significance level: ***$p \le 0.001$

Series of stride durations of a healthy adult acquired during a pilot test and of a representative patient with Parkinson’s disease in T0 and T1 are shown in Fig. 2. As expected, the LRA level, i.e., $\alpha$ exponent, is lower for the patient than for the healthy adult. It can also be noted that the mean stride duration of the patient decreased from T0 to T1. Figure 3 reports the hip angle profile of a representative patient. It can be observed that the ROM is larger in T1 and T2 than in T0.

These representative trends were further assessed at the population level by running statistical tests. Assessment of spatiotemporal gait metrics (Fig. 4a–c) indicate an increase in gait speed and stride length and a decrease in stride duration between T0 and T1 ($p < 0.001$) and T0 and T2 ($p < 0.001$). The hip ROM (Fig. 4d) also increased from T0 to T1 ($p < 0.001$) and to T2 ($p < 0.001$). Note that linear mixed-effects models are accounting for individual biases via the term capturing random intercepts in Eq. (3). Statistical tests are therefore robust even if some subjects deviate from the group average.

In contrast, no significant difference was found in the mean level of LRA in the inter-stride time series, indicated by the $\alpha$ exponent, between evaluation sessions (Fig. 5). However, the inter-subject variance in LRA exponent during T1 was significantly lower than in T0 ($p < 0.01$) and in T2 ($p < 0.05$). Concerning the individual evolution of this $\alpha$ exponent between T0 and T1, five subjects with lower initial LRA levels had a mean increase of 16% (#2, #3, #5, #6, #8), while the three others had a mean decrease of 8% (#1, #4, #7), as shown in Fig. 5.

Regarding the behavior of the orthosis during the training sessions (Fig. 6), the maximal torque and energy injected at the hip significantly decreased across training sessions ($p < 0.001$ for both metrics).

Finally, the ABC score was significantly higher in T1 and T2 compared to T0 ($p < 0.05$), with a mean ± SD score of 35.63 ± 9.64 (maximum possible is 45) in T0, 37.88 ± 8.01 in T1 and 38.25 ± 7.15 in T2. In contrast, no significant difference was found in the other clinical metrics, i.e., neither in the MDS-UPDRS part III score, even when divided into its Postural Instability and Gait Difficulty and rigidity subscores, nor in the Mini-BESTest score.

## Discussion

Numerous studies have shown the beneficial effects of robot-assisted gait training, divided into 10–20 sessions of 25–40 min over 4–5 weeks as reviewed by [21], for improving spatiotemporal gait metrics in patients with Parkinson’s disease. They particularly showed an increase in gait speed, stride length and cadence [7–13, 15–19]. These three metrics are connected since the increase in gait speed can be enhanced by increasing cadence, stride length, or both [13]. These results are in accordance with those of the present study showing an increase in gait speed, stride length and cadence—equivalent to the observed decrease in stride duration—, and we further showed that these positive outcomes are maintained one month after the end of the training. Several hypotheses have been raised by previous papers to explain these positive evolutions after training with robotic devices. First, Sale and colleagues [15] suggested that these improvements were due to the intense repetition of a stereotyped gait pattern, which induced somatosensory cueing and stimulation. Ustinova and co-workers [8] also stated that improvements of these spatiotemporal gait metrics were due to the use of the treadmill, being necessary with the Lokomat exoskeleton, building upon results from other studies using a treadmill alone. Nevertheless, the present study tends to show that it is possible to obtain equivalent results after overground gait training with a compliant orthosis that does not follow a stereotyped gait pattern. We rather explained these improvements in gait parameters by the increased ROM, which, to the best of our knowledge, has never been reported in previous studies. This increase could be due to the assistance provided by the robot that compensates for a disease-induced hip flexor muscle weakness [54]. Observing this result is facilitated by the semi-ecological environment used in our study, since the patients’ hips kinematics were constrained neither by the environment nor by the provided assistance. We hypothesize that this larger hip ROM helped patients to increase their cadence and stride length, and therefore their gait speed. Interestingly, these changes in gait occurred even if the maximal injected torque was moderate (about 0.1 Nm/kg, i.e., about 17% of what a healthy hip delivers during overground walking [55]), and this torque moreover decreased along training sessions. These improvements are very important in preventing falls for patients with Parkinson’s disease. Indeed, a decrease in these gait metrics is considered as a marker of a higher risk of falling [3]. An important caveat to this discussion is that similar results could have been observed after an equivalent amount of exercising without the robot. This was not addressed in this study, since no control group was included. Nevertheless, several studies involving control groups performing conventional physiotherapy (i.e., joints mobilization, conventional overground gait training, muscle stretching,...) with the same intensity as a robot-assisted group reported larger effects with the latter as compared to the former group [13, 14, 16]. It is also interesting to mention that some patients spontaneously reported that being assisted by a robot helped them and increased their motivation. Indeed, some patients arrived at the training session being tired, and the robotic assistance encouraged them to carry on with the session until the end.

Regarding the clinical metrics, only the balance confidence (ABC scale) decreased after training, and this result was maintained after 1 month post-training. This result was also reported in previous articles [14, 56], and was associated with an improvement in balance functions. Similar improvements in balance were not identified in our results through the Mini-BESTest. Since the ABC scale is a subjective one, this result shows that patients felt an improvement in their self-perceived balance confidence after this robot-assisted gait training, although this was not confirmed by a measured improvement in their postural control assessed with the Mini-BESTest score. This can be explained by the fact that both studies reporting increased balance functions involved patients in more advanced stages (H&Y 2.5–4), thus having more pronounced postural instability than those of the present study. Another potential explanation for the lack of balance improvement in this study is the absence of body weight support, in contrast to previous studies reporting an improvement in this parameter. With body weight support, it was hypothesized that patients can better regulate weight shifting during walking [14, 57]. On the other hand, the scale rating the motor symptoms did not improve either. This is probably because training with the orthosis was only intended to impact the patients’ gait, and not other motor aspects of the disease assessed by the MDS-UPDRS part III scale, such as rigidity, bradykinesia, or tremor [56].

Finally, the level of LRA in series of stride durations of patients with Parkinson’s disease was $0.66 \pm 0.11$ before training (Fig. 5), which is lower than the one of healthy walkers, i.e., $0.82 \pm 0.04$ as computed by applying AFA on 10 series of 1024 strides from [53]. Having a decreased LRA level in series of stride durations indicates a more random temporal organization of the series, which is thought to be a marker of gait instability in pathological populations [32]. However, in the present study, the level of LRA of patients did not significantly increase after the training sessions; although individual data were more clustered around a value of $\alpha$ exponent closer to the one of healthy individuals. Indeed, the five subjects who displayed the lowest level of LRA before training (T0) increased it during the second evaluation session (T1). In contrast, this level slightly decreased or remained constant for the three participants who had a high level before training. These levels returned to, or exceeded, their initial values in T2, indicating that there was no training retention effect after 1 month. The models described in [36, 37] predicted that the level of LRA in series of stride durations should increase when the subject is assisted by the device. The present results suggest that a training with the device standardized this level in patients with Parkinson’s disease, by increasing it for patients who had a lower initial one. Further investigations should be conducted to assess the potential rehabilitative effect of this observation, and the consequence of the fact that it is not retained in the longer term.

We did not find a relationship between the variation in the level of LRA and other metrics assessed in this study. In particular, no correlation has been found between the $\alpha$ exponent and the H&Y score, reflecting the level of disease progression. This may be because this study mostly included patients with a moderate disease stage (H&Y 2$-$2.5), and is therefore not capturing the whole spectrum of gait impairments encountered in patients with Parkinson’s disease. Further experiments should be conducted on a wider range of stages and on a larger number of patients to identify whether a specific stage of the disease would better respond to this therapy. Moreover, this difference across patients’ response to robot-assisted gait training can have other origins than motor functions as assessed by the H&Y scale. Indeed, because of the heterogeneity of Parkinson’s disease, every patient is not impacted in the same way by the disease. There is a large variability in symptoms and disease progression across individuals. This is due for example to genetic factors causing patients to respond differently to the same drug [58], or to a more active lifestyle slowing down the disease progression [59]. All these differences have led clinicians to create different sub-groups of patients, based on age of onset, motor phenotype, nonmotor symptoms and genetic mutations. This heterogeneity of the disease further emphasizes the importance of personalized treatment for each patient [60]. The present study suggests that robot-assisted gait training might lead to different effects regarding LRA as a function of the patient profile. Further investigations should be conducted to establish if this is connected to genetic or behavioral markers.

Despite the small sample size of the present study, these experiments highlighted interesting results for mitigating gait disorders in patients with Parkinson’s disease. A larger and more diversified sample (in terms of H&Y stage and gender diversity) could help to show an improvement in the level of LRA in series of stride durations of these patients. Moreover, a longer training period, or incorporating this device into weekly physiotherapy sessions, might also induce an improvement in this metric, and potentially longer-term retention after training.

## Conclusion

This study showed that an adaptive walking assistance delivered by a wearable robot does improve several gait metrics in patients with Parkinson’s disease, such as gait speed, stride duration and length, and hip ROM. It also opened new research avenues for assessing the effects of such assistance on the level of LRA in series of stride durations, in order to identify which patient profile might benefit the most of this assistance, especially regarding this particular motor control metric.

## Data Availability

The data that support the findings of this study are available from Össur hf. (Reykjavik, Iceland) but restrictions apply to the availability of these data, which were used under license for the current study, and so are not publicly available. Data are however available from the authors upon reasonable request and with permission of Össur hf.

## Abbreviations

AFA: Adaptive fractal analysis
APO: Active pelvis orthosis
H&Y: Hoehn and Yahr score
LRA: Long-range autocorrelations
ROM: Range of motion
